# Supplementary material for: Atlantic Salmon (Salmo salar) Transfer to Seawater by Gradual Salinity Changes Exhibited an Increase in The Intestinal Microbial Abundance and Richness
Source: Microorganisms. 2022 Dec 27;11(1):76. doi: 10.3390/microorganisms11010076 (PMC9865641; doi:10.3390/microorganisms11010076)
Supplement: Supplementary file 1 [file microorganisms-11-00076-s001.zip › SuppInfo_Figures.pdf]

Supplementary Figures

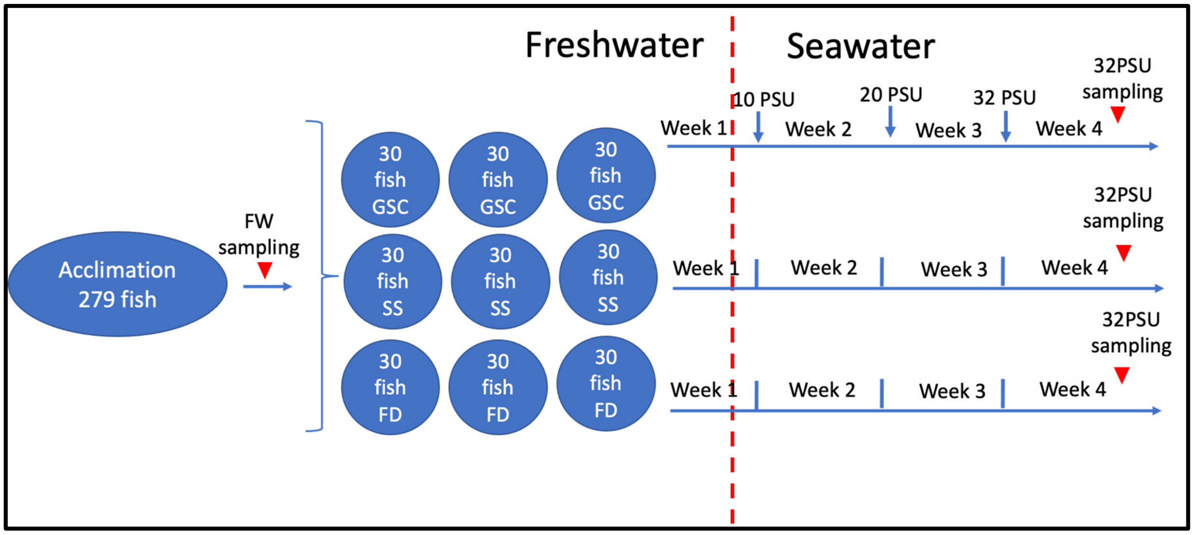

Figure S1. Experimental design for the Atlantic salmon transfer from FW to SW. After a month, all fish were acclimated in the same tank and were separated into three experimental groups: Gradual salinity change (GSC), salinity sock (SS), and functional diet (FD). The blue arrows indicate the salinity change performed in the GSC group. Sample points are indicated with a red arrow.

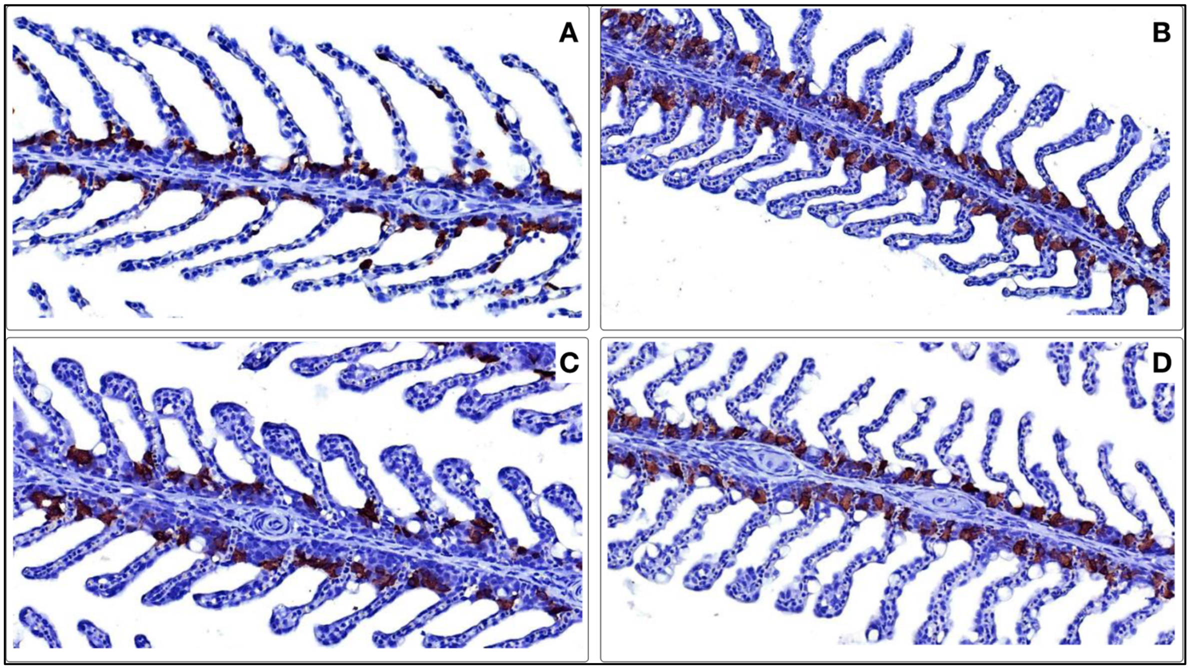

Figure S2. Localization of chloride cells in the gill filament epithelium. (**A**) Freshwater (FW) gills sample; (**B**) Gradual salinity change (GSC) 32 PSU; (**C**) Salinity shock (SS) 32 PSU; D) functional diet (FD) 32 PSU. A) Na+/K+-ATPase positive cells located in the middle region of the superficial interlamellar space and near the lamellar vascular axis. B-C Na+/K+-ATPase positive cell deeper in the epithelium.

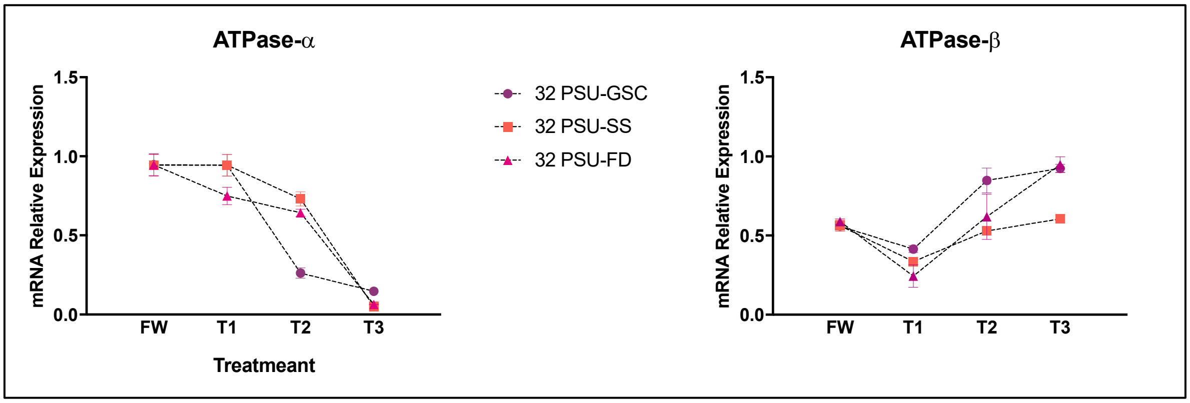

Figure S3. RT-qPCR analysis of ATPase- α and ATPase-β subunits. FW: Freshwater previous treatment group; GSC: gradual salinity change at 10 PSU, 20 PSU, and 32 PSU groups; 32PSU-SS Salinity shock at 32-PSU group; 32PSU-FD: Salinity shock at 32 PSU group previously feeding with a functional diet.

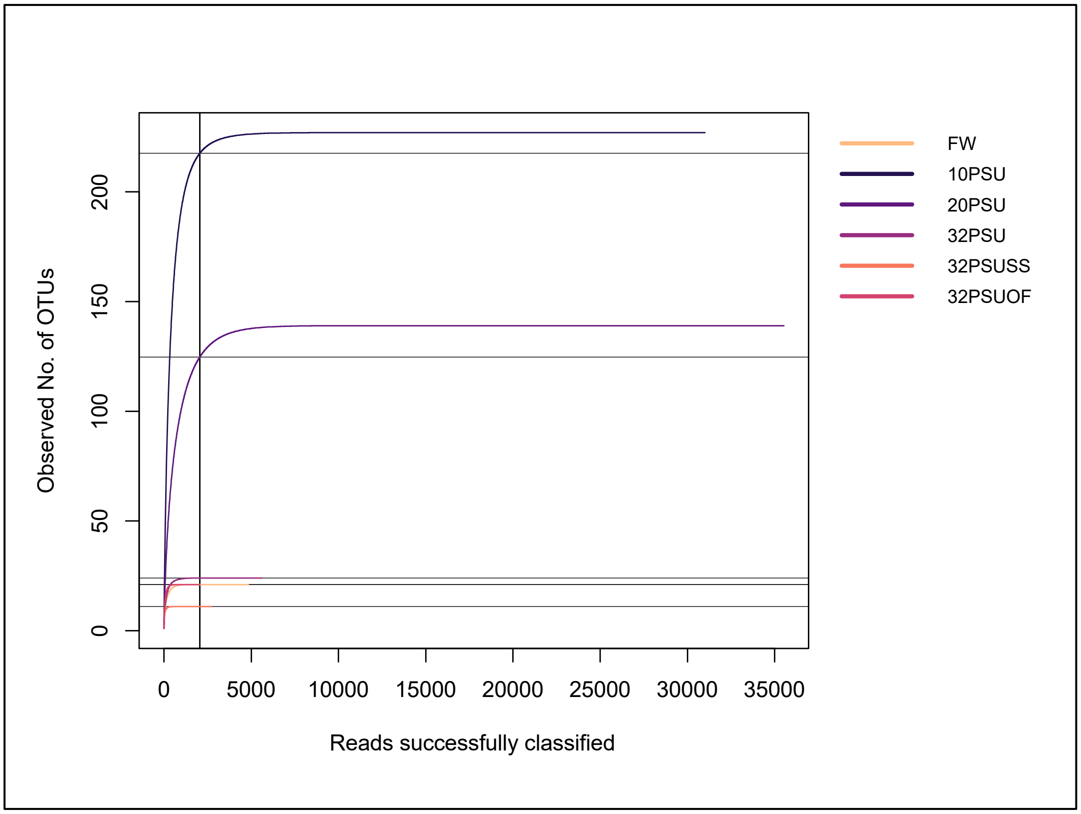

Figure S4. Rarefaction curve. FW: Freshwater previous treatment group; GSC: gradual salinity change at 10 PSU, 20 PSU, and 32 PSU groups; 32PSU-SS Salinity shock at 32-PSU group; 32PSU-FD: Salinity shock at 32 PSU group previously feeding with a functional diet.

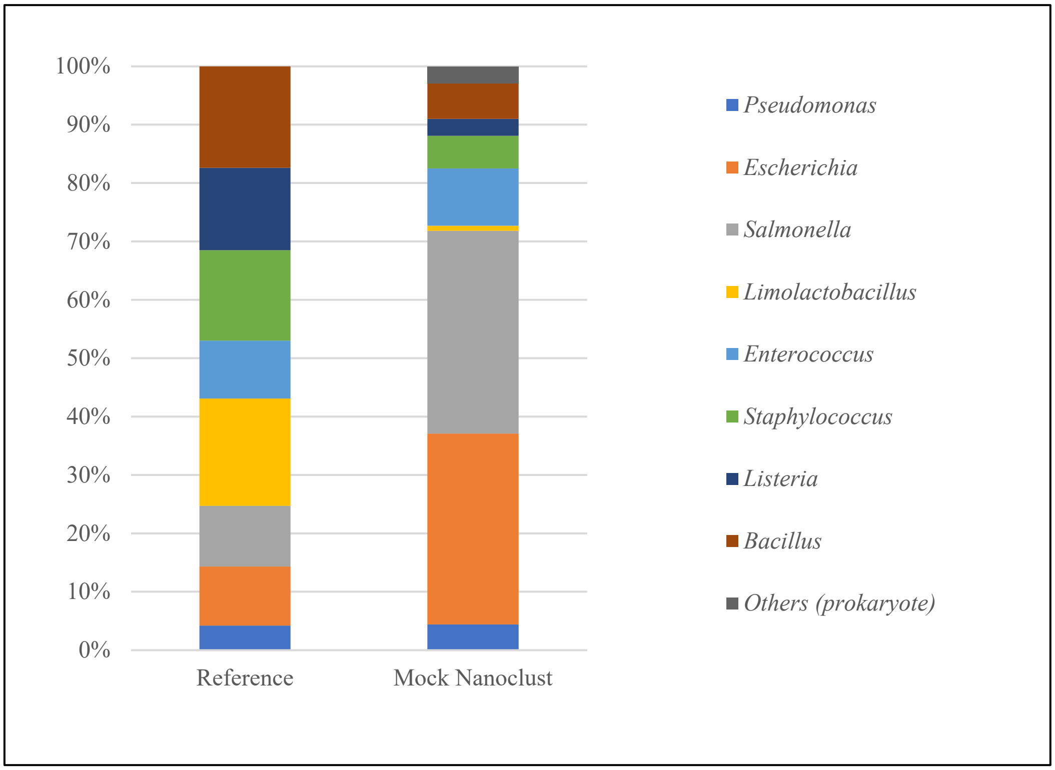

Figure S5. Mock community composition
