# Supplementary material for: Atlantic Salmon (Salmo salar) Transfer to Seawater by Gradual Salinity Changes Exhibited an Increase in The Intestinal Microbial Abundance and Richness
Source: Microorganisms. 2022 Dec 27;11(1):76. doi: 10.3390/microorganisms11010076 (PMC9865641; doi:10.3390/microorganisms11010076)
Supplement: Supplementary file 1 [file microorganisms-11-00076-s001.zip › SuppInfo_Table_S1.pdf]

Table S1. Sequencing data report

PycoQC sequencing report					
**Run**	**Reads**	**Pass reads**	**Bases**	**N50 length**	**Med Read Quality**
Run1	1.057.010	572.635	233.406.176	477	8,15
Run2	3.687.103	2.513.372	1.100.488.945	434	8,76
Run3	4.077.332	2.781.903	1.220.612.486	444	8,99
Total	8.821.445	5.867.910	2.554.507.607		

Classification report by treatment			
	**Pass Porechop**	**Pass NanoCLUST**	**Pass NanoCLUST classification**
Mock	723.496	13.162	11.527
FW	406.455	5.139	4.920
10 PSU-GSC	198.492	38.488	30.835
20 PSU-GSC	150.384	49.368	35.524
32 PSU-GSC	393.174	8.163	5.594
32 PSU-FD	553.295	2.180	2.051
32 PSUSS	285.080	3.047	2.709
Total	2.710.376	119.547	93.160

FW: Freshwater previous treatment group, GSC: gradual salinity change at 10 PSU, 20 PSU and 32 PSU groups; 32PSU-SS Salinity shock at 32-PSU group; 32PSU-FD: Salinity shock at 32 PSU group previously feeding with a functional diet.
