# Supplementary material for: Atlantic Salmon (Salmo salar) Transfer to Seawater by Gradual Salinity Changes Exhibited an Increase in The Intestinal Microbial Abundance and Richness
Source: Microorganisms. 2022 Dec 27;11(1):76. doi: 10.3390/microorganisms11010076 (PMC9865641; doi:10.3390/microorganisms11010076)
Supplement: Supplementary file 1 [file microorganisms-11-00076-s001.zip › SuppInfo_Table_S3.pdf]

Table S3. Bray-Curtis similarities for intestine samples calculated on R with Vegan Package.

	FW	10PSU-GSC	20PSU-GSC	32PSU-GSC	32PSU-SS
**10PSU-GSC**	0.9122202				
**20PSU-GSC**	0.8767837	0.6564537			
**32PSU-GSC**	0.6864566	0.9405967	0.8746218		
**32PSU-SS**	0.8784126	0.9771927	0.9612655	0.6726891	
**32PSU-FD**	0.8491341	0.9768470	0.9432824	0.7866374	0.7323160

FW: Freshwater previous treatment group, GSC: gradual salinity change at 10 PSU, 20 PSU, and 32 PSU groups; 32PSU-SS Salinity shock at 32-PSU group; 32PSU-FD: Salinity shock at 32 PSU group previously feeding with a functional diet.
