# Supplementary material for: Atlantic Salmon (Salmo salar) Transfer to Seawater by Gradual Salinity Changes Exhibited an Increase in The Intestinal Microbial Abundance and Richness
Source: Microorganisms. 2022 Dec 27;11(1):76. doi: 10.3390/microorganisms11010076 (PMC9865641; doi:10.3390/microorganisms11010076)
Supplement: Supplementary file 1 [file microorganisms-11-00076-s001.zip › SuppInfo_Table_S4.pdf]

Table S4. NanoCLUST average percent identity of Clusters at the species level. Species with percentage of identity ≥ 98.7 based on the full sequence of 16S rRNA gene and relative abundance ≥ 0.2%.

	Average identity (%)±SD					
**Species identifies by NanoCLUST**	**FW**	**10PSU-GSC**	**20PSU-GSC**	**32PSU-GSC**	**32PSU-SS**	**32PSU-FD**
Acidovorax radicis			99.2±0.00			
*Acinetobacter johnsonii**	95.2±3.67	95.3±3.85	91.5±0.00	93.4±2.29	99.3±0.00	99.2±0.00
Akkermansia muciniphila		99.2±0.00				
*Aliivibrio wodanis**				99.0±0.00		
Alistipes onderdonkii		91.6±3.15	99.0±0.00			
Alistipes putredinis		94.1±7.42	99.8±0.00			
Anaerosinus glycerini			99.4±0.00			
Arcobacter venerupis		99.6±0.00				
Bacillus cereus	99.1±0.00					99.8±0.00
Bacteroides caccae		99.4±0.00				
Bradyrhizobium mercantei	99.0±0.00					
Bradyrhizobium rifense		99.4±0.00				
Cloacibacterium caeni		99.5±0.00				
Cloacibacterium normanense			99.4±0.00			
Cutibacterium acnes		99.7±0.00	99.9±0.00			
Elizabethkingia anophelis						99.0±0.00
Eubacterium rectale		99.5±0.00				
Flavobacterium cheniae		99.3±0.00				
*Flavobacterium succinicans**		98.1±7.25	98.8±4.22			
Heliimonas saccharivorans						98.9±0.00
Herbaspirillum huttiense						99.0±0.00
Lactococcus raffinolactis		93.0±0.49	99.5±0.00			
Megasphaera massiliensis		99.1±0.00				
Methylobacterium brachiatum				99.1±0.00		
Methylobacterium radiotolerans	97.5±0.00					
Microbacterium ginsengisoli	99.6±0.00	99.8±0.00				

Microbacterium mangrovi				94.6±0.00		
Micrococcus luteus				99.7±0.00		
Moraxella osloensis	98.6±0.00	98.6±0.00		99.3±0.00	99.0±0.00	
Morganella morganii		97.6±1.60	98.7±0.00			
Paraburkholderia fungorum					98.5±0.00	99.2±0.00
Pelomonas saccharophila						99.1±0.00
Phascolarctobacterium succinatutens		99.0±0.00				
Phocaeicola coprocola		99.2±0.00				
*Providencia rettgeri**		98.9±0.00				
Pseudomonas brenneri		99.4±0.00				
Pseudomonas cedrina						99.4±0.00
Pseudomonas migulae	98.5±0.00	99.1±0.00	95.5±0.00	99.3±0.00		
Rahnella aquatilis		99.2±0.00				
Ralstonia insidiosa						99.4±0.00
Rhizobium zeae	99.6±0.00	99.4±0.00				
Shigella flexneri	97.3±2.47	99.5±0.00	97.0±3.38	95.9±0.34		
Shigella sonnei			93.5±0.00	98.9±0.00		
Sphingobium yanoikuyae				99.5±0.00		
Staphylococcus aureus			99.6±0.00	96.9±0.00		
Streptococcus thermophilus	99.8±0.00		99.5±0.00	98.9±0.00		
Thermosipho melanesiensis						100.0±0.00
Uruburuella suis		99.6±0.00				

SD: Standard deviation; *indicate fish-pathogenic bacteria. FW: Freshwater previous treatment group, GSC: gradual salinity change at 10 PSU, 20 PSU and 32 PSU groups; 32PSU-SS Salinity shock at 32-PSU group; 32PSU-FD: Salinity shock at 32 PSU group previously feeding with a functional diet.
